# A case report of an isolated superior mesenteric artery dissection caused by childbirth

**DOI:** 10.1186/s12876-021-01994-0

**Published:** 2021-11-13

**Authors:** Qian Feng, Jingrun Zhao, Lina Zang, Yuanyuan Chen, Senlin Li

**Affiliations:** Department of Gastroenterology, Liaocheng People’s Hospital, Liaocheng, China

**Keywords:** Isolated superior mesenteric artery dissection, Abdominal pain, Delivery, Case report

## Abstract

**Background:**

The isolated superior mesenteric artery dissection (SMAD) is a rare and sporadic cause of acute abdominal pain. It most frequently affects male patients in their fifth to sixth decades, while our patient was a young woman who delivered a baby before the onset of abdominal pain. Possible risk factors for SMAD include hypertension, arteriosclerosis, abnormalities in elastic fibres, trauma, and pregnancy. In our case, delivery was suggested as a risk factor, which has not been reported previously.

**Case presentation:**

A 27-year-old woman complained of acute severe upper abdominal pain and vomiting for 2 days after delivery. The patient had no significant medical history. Physical examination revealed epigastric mild tenderness. All routine blood tests, blood coagulation analysis, liver function tests and abdomen computed tomography showed no remarkable findings. Computed tomography angiography revealed a marked dissection 3.5 cm below the superior mesenteric artery ostium. Since distal blood flow existed and the patient was in a puerperal state with no evidences of mesenteric ischemia, she was managed conservatively, including intestinal rest by fasting, parenteral nutritional support and antibioticis, without anticoagulants or antiplatelet agents. Fortunately, she recovered smoothly and had no recurrence.

**Conclusions:**

SMAD is a rare and sporadic cause of acute abdominal pain that occurs in young women after delivery.

## Background

Isolated superior mesenteric artery dissection (SMAD) has been anecdotally reported as a rare and sporadic cause of acute abdominal pain, with an incidence of approximately 0.06% according to postmortem investigations [1]. The majority of SMAD patients have been reported to be middle-aged men [2]. Treatment options for SMAD include conservative therapy, endovascular repair, and surgery. Here, we report a case of SMAD in a young woman after childbirth who was cured with conservative medical managements without anticoagulant therapy.

## Case presentation

**Fig. 1:**
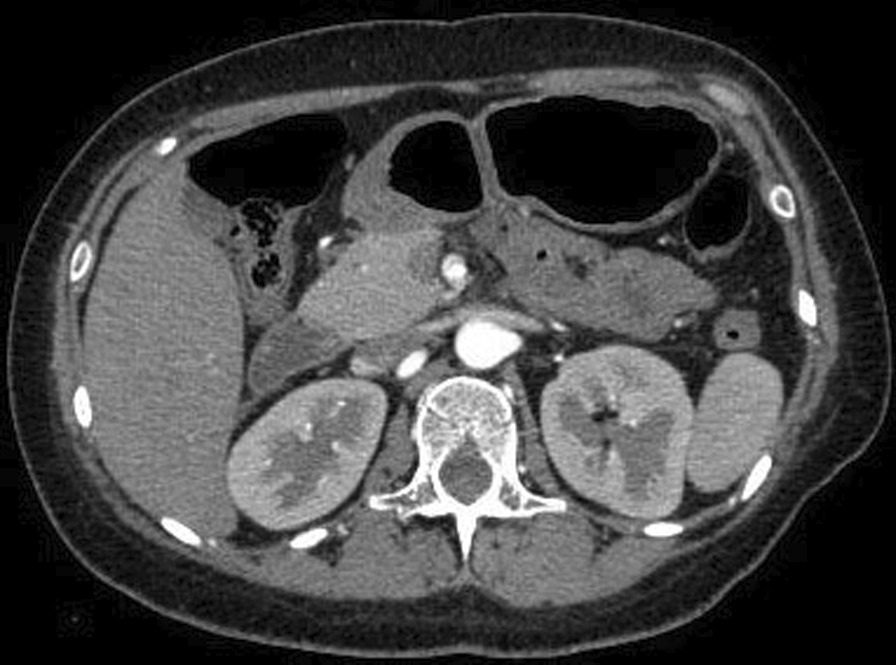
Computed tomography angiography (CTA) demonstrated a marked superior mesenteric artery dissection

**Fig. 2:**
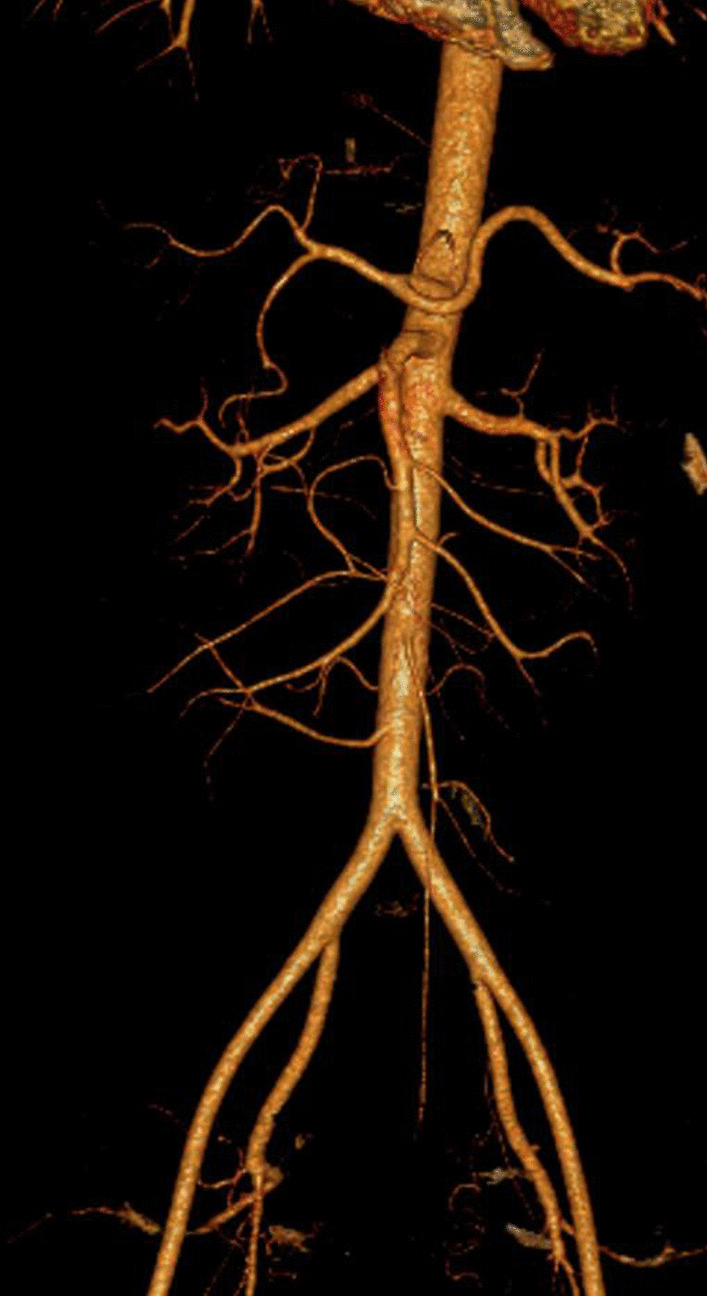
The location of the SMAD was 3.5 cm down of the superior mesenteric artery ostium

A 27-year-old woman was transferred to our hospital in October 2015 complaining of acute severe upper abdominal pain for 2 days after delivery of a baby, which was accompanied by vomiting and could not be relieved by acid suppression and antispasmodic treatments. The patient had no smoking habits, no hypertension history, no significant medical and family history except for delivery 2 days prior, which was not smooth and lasted for 23 h. Oxytocin was applied and forceps was not used. Hypertension peak during delivery was 130/80 mmHg. Physical examination revealed epigastric mild tenderness without any signs of peritonitis. All routine blood tests, blood coagulation analysis, liver function tests and computed tomography showed no remarkable findings. Thus, computed tomography angiography (CTA) was performed for further evaluation, which revealed a marked SMAD (Fig. 1) 3.5 cm below the superior mesenteric artery (SMA) ostium (Fig. 2). Therefore, the SMAD diagnosis was confirmed. The SMA-distal aorta angle was 85.5°. The distal blood flow was preserved via the mesenteric marginal artery, and bowel infarction did not occur. Considering that the patient was in a puerperal state and still had vaginal bleeding, we managed her conservatively, including intestinal rest by fasting, antibiotic therapy and parenteral nutrition, without anticoagulants or antiplatelet agents. Fortunately, abdominal pain was gradually alleviated, and she was able to consume liquid food 3 days after admission. Her symptoms were not exacerbated by eating. We followed up for 48 months during which no recurrence occurred.

## Discussion and conclusions

Since SMAD was first described by Bauersfeld [3] in 1947, an increasing number of cases have been reported owing to advances in imaging technology. However, because of the rarity of this disease, the underlying causes and treatments have not been determined.

A higher oscillatory shear index has been observed at the anterior wall of the SMA near the convex curvature with a larger SMA-distal aorta angle, where SMAD commonly occurs, suggesting mechanical stress as a putative cause [4–6]. The angle of our patient was 85.5°, much larger than 38° to 60° of normal individuals [7]. Possible risk factors for SMAD include hypertension, arteriosclerosis, abnormalities in elastic fibres, trauma, pregnancy and genomic alterations [8–10]. In this case, we considered unsmooth delivery as the chief predisposing cause, which could not only weaken the arterial wall but also cause sudden abdominal hyperpressure, followed by further mechanical stretching and microtrauma of the SMA. In addition, fat consumption by the patient could increase the burden on the bowel and aggravate abdominal pain. Unlike the other 88% of cases in which SMAD occurred in men with a mean age of 54 years [2], our patient was a young woman who delivered a child 2 days prior. In our case, the distance between the SMA ostium and the origin of the SMAD was 3.5 cm, which was similar to the range of 3–4 cm in other reports [11]. According to the Sakamoto classification, the true lumen residual diameter and severity of narrowing in the true lumen were respectively 0.4 cm, 40% in our case.

SMAD presents with abdominal pain in most cases. Some researchers have indicated that the severity of abdominal pain was correlated with the length of dissection [12]. Other associated signs include vomiting, haematochezia, abdominal distension, and fever. The major complications of SMAD are arterial rupture with bleeding, acute mesenteric ischemia (AMI) [13], bowel infarction [14–16] and progressive dilatation of the SMA [17]. AMI is the most feared complication to expect and to monitor. Otsuka et al. [18] have reported AMI should be diagnosed when the following findings were present: unrelieved abdominal pain, an increased lactate or D-dimer level, and CT characteristics of bowel wall thinning, decreased bowel wall enhancement and bowel loop diameter > 2.5 cm [19].

Ultrasonography, contrast computed tomography (CT), CTA, MRI and angiography can all be used to diagnose SMAD; CTA [13, 20] is preferred and recommended first to rule out small bowel ischemia and/or infarction.

The management relies in: (1) conservative therapies including bowel rest, blood pressure control, oral/enteral antibiotics and parenteral nutritional support, with or without antithrombotic therapy; (2) the revascularization of viable intestine and (3) the surgical resection of necrotic bowel, based on symptoms, comorbidities, anatomic suitability, and physician’s preference [13]. The administration of systematic oral/enteral antibiotic therapy finds its justification in: (1) the limitation of microbial proliferation in contact with ischemic intestinal lesions; (2) prevention of microbial translocation; (3) the low bioavailability of antibiotic administered intravenously at the ischemic site. Controversy existed regarding the role of antithrombotic use. But SMAD could progress to AMI and the distal flow may be possibly reduced because of luminal narrowing except for SMAD of type I (Sakamoto classification) [17]. Thus, some physicians prefer routine or selective antithrombotics [21], while Peng et al. suggested anticoagulants should be started in the absence of clinical improvement [22]. Meanwhile, there is no consensus on the timing of interventional therapy or surgery. Park et al. [23] suggested that intervention should be performed if abdominal pain lasted over 7 days, whereas Jia et al. [24] recommended endovascular repair if symptoms persisted for more than 24 h. Endovascular stenting can be used as a primary treatment option for symptomatic patients with severe stenosis and failed conservative treatments [25]. Surgery may be required following published risk factors of irreversible intestinal necrosis including organ failure (defined as a score ≥ 2 in any system of the Marshall score [26]), elevated serum lactate (> 2 mmol/l) as well as bowel loop dilation (diameter > 2.5 cm) on CT [19]. In our case, considering the young age of the patient, vaginal bleeding, no sign of bowel necrosis and moderate stenosis of the SMA, we chose conservative treatment without antithrombictics and achieved satisfactory curative effects.

The research [22] from Mayo clinic reported recurrence rates as high as 20% which may lead to life-threatening mesenteric ischemia and thus necessitates appropriate surveillances.

In conclusion, we report a case of SMAD that occurred in a young woman after childbirth and was cured by conservative means. Since SMAD can be recurrent [4], regular follow-up is essential. No recurrence was detected at the last follow-up of 48 months.

## Data Availability

All data are included in this article and its supplementary information files.

## Abbreviations

SMAD: Superior mesenteric artery dissection
CTA: Computed tomography angiography
SMA: Superior mesenteric artery
CT: Computed tomography
AMI: Acute mesenteric ischemia
